# Knowledge, Health Seeking Behavior and Perceived Stigma towards Tuberculosis among Tuberculosis Suspects in a Rural Community in Southwest Ethiopia

**DOI:** 10.1371/journal.pone.0013339

**Published:** 2010-10-11

**Authors:** Gemeda Abebe, Amare Deribew, Ludwig Apers, Kifle Woldemichael, Jaffer Shiffa, Markos Tesfaye, Alemseged Abdissa, Fetene Deribie, Chali Jira, Mesele Bezabih, Abraham Aseffa, Luc Duchateau, Robert Colebunders

**Affiliations:** 1 Department of Medical Laboratory Sciences and Pathology, Jimma University, Jimma, Ethiopia; 2 Department of Epidemiology, Jimma University, Jimma, Ethiopia; 3 Department of Clinical Sciences, Institute of Tropical Medicine, Antwerp, Belgium; 4 Department of Internal Medicine, Jimma University, Jimma, Ethiopia; 5 Department of Psychiatry, Jimma University, Jimma, Ethiopia; 6 Department of Health Service Management, Jimma University, Jimma, Ethiopia; 7 Armauer Hanssen Research Institute, Addis Ababa, Ethiopia; 8 Department of Comparative Physiology and Biometrics, University of Ghent, Ghent, Belgium; 9 Department of Epidemiology and Social Medicine, University of Antwerp, Antwerp, Belgium; St. Petersburg Pasteur Institute, Russian Federation

## Abstract

**Background:**

Perceived stigma and lack of awareness could contribute to the late presentation and low detection rate of tuberculosis (TB). We conducted a study in rural southwest Ethiopia among TB suspects to assess knowledge about and stigma towards TB and their health seeking behavior.

**Methods:**

A community based cross sectional survey was conducted from February to March 2009 in the Gilgel Gibe field research area. Any person 15 years and above with cough for at least 2 weeks was considered a TB suspect and included in the study. Data were collected by trained personnel using a pretested structured questionnaire. Logistic regression analysis was done using SPSS 15.0 statistical software.

**Results:**

Of the 476 pulmonary TB suspects, 395 (83.0%) had ever heard of TB; “evil eye” (50.4%) was the commonly mentioned cause of TB. Individuals who could read and write were more likely to be aware about TB [(crude OR = 2.98, (95%CI: 1.25, 7.08)] and more likely to know that TB is caused by a microorganism [(adjusted OR = 3.16, (95%CI: 1.77, 5.65)] than non-educated individuals. Males were more likely to know the cause of TB [(adjusted OR = 1.92, (95%CI: 1.22, 3.03)] than females. 51.3% of TB suspects perceived that other people would consider them inferior if they had TB. High stigma towards TB was reported by 199(51.2%). 220 (46.2%) did not seek help for their illness. Individuals who had previous anti-TB treatment were more likely to have appropriate health seeking behavior [(adjusted OR = 3.65, (95%CI: 1.89, 7.06)] than those who had not.

**Conclusion:**

There was little knowledge about TB in the Gilgel Gibe field research area. We observed inappropriate health seeking behavior and stigma towards TB. TB control programs in Ethiopia should educate rural communities, particularly females and non-educated individuals, about the cause and the importance of early diagnosis and treatment of TB.

## Introduction

TB is a major cause of illness and death worldwide. The burden is rising globally as a result of poverty, population growth and HIV/AIDS [1], [2]. The World Health Organization (WHO) reported 9.27 million cases of TB in 2007. Among the 15 countries with the highest estimated TB incidence rates, 13 are in Africa which accounts for 31% of the global total [3].

In Ethiopia, TB is the major cause of death and hospital admission [4]. The country stands 7^th^ among the 22 highest burden countries [3]. The Directly Observed Treatment Short course (DOTS), the internationally recommended strategy for TB control, was adopted in 1992. DOTS comprises five components of which case detection by sputum smear microscopy and standardized treatment with supervision and patient support are the major ones[5]. Despite the early introduction of DOTS, case detection rate of smear positive pulmonary TB is still very low as compared to the 70% target recommended by WHO [4].

The low case detection rate could be attributed to many factors which could be broadly categorized as patient related delay of health care seeking or failure of the health care system to diagnose patients. Studies have reported that patient delay represented 77% of the total delay period from onset of symptoms to initiation of treatment [6], [7].

The DOTS strategy depends on the self presentation of patients to the health centers. Moreover, its success in case finding depends on patient motivation, degree of diagnostic suspicion by health care workers, and the accuracy and effectiveness of diagnostic laboratory services [8]. Delay in diagnosis may lead to progression of disease leading to increased mortality and enhanced TB transmission in the community. Reports have indicated that patients become more contagious as the delay progresses [9]. Treatment delay, which mainly results from delayed diagnosis, is one of the major challenges of TB control programs in developing countries [10], [11], [12]. It is particularly important in a high HIV prevalence setting like Ethiopia [13], [14] where persons with HIV infection are at high risk of developing TB.

Studies in different countries report that knowledge about and stigma towards TB is affected by socio-economic variables [15], [16], [17]. Low awareness[18], [19], [20], stigma [21], [22], income [23], rural residence [18], illiteracy [18], gender [20], [24], [25], marital status [19] and distance to the clinic [10], [19], [25] were reported to affect the health care seeking behavior [26], [27].

Most studies about knowledge and stigma towards TB and health seeking behavior were done among patients seeking medical care in health institutions [18], [24], [26]. The present study was done in rural southwest Ethiopia among TB suspects to describe the knowledge about and stigma towards TB and how that knowledge and stigma affected their health-seeking behavior.

## Methods

### Study Design and Area

A community based cross sectional survey was conducted from February to March 2009 in the Gilgel Gibe field research area, in southwest Ethiopia. The Gilgel Gibe field research area is located in Jimma Zone about 260 km southwest of Addis Ababa around the reservoir of Gilgel Gibe hydroelectric dam. The site is bounded by four districts: Sokoru, Omo-Nada, Tiro-Afata and Qarsa. In the four districts, two small towns and eight rural Kebeles (smallest administrative units), which are found within 10 kilometers of the reservoir of the dam were selected as field research area by Jimma University in 2005. Since 2005, demographic and AIDS mortality surveillance has been undertaken by Jimma University. At the time of the study, the total population of the field research area was 50,156 individuals from 10,859 households.

### Study Population

The study population consisted of all adult TB suspects (described in Table 1) in the Gilgel Gibe Field Research area during the study period. The proportion of TB suspects among the adult population was 2.6% in 2005 [28] and 1.4% in 2002 [29] in two separate studies in southern and central parts of Ethiopia respectively. Using the mean of these two studies (2%), the estimated number of TB suspects among 25,000 adults in the field research area was 500. To find the 500 TB suspects, all households in the research area were visited. Any person with cough for at least 2 weeks was considered as a TB suspect. Individuals less than 15 years of age or temporary residents were excluded from the study. From each TB suspect two sputum samples were collected and analyzed by AFB microscopy. AFB positive study participants were counseled about TB and linked to the nearest health centre to start anti-TB treatment.

**Table 1:** Socio-demographic characteristics of the TB suspects (n = 476) in the Gilgel Gibe field research area, south west Ethiopia, March 2009.

Characteristic	Number (%)
**Sex**	
Male	189 (39.7)
Female	287 (60.3)
**Age, mean (SD)**	40.9 (16.2)
**Marital status**	
Married	347 (72.9)
Single	50 (10.5)
Divorced	26 (5.5)
Widowed	53 (11.1)
**Ethnicity**	
Oromo	435 (91.4)
Amhara	10 (2.1)
Yem	20 (4.2)
Others	11 (2.3)
**Religion**	
Muslim	431 (90.5)
Orthodox	40 (8.4)
Protestant	5 (1.1)
**Education**	
Had formal education	82 (17.2)
No formal education	394 (82.8)
**Occupation**	
Farmer	308 (64.7)
Housewives	70 (14.7)
Student	20 (4.2)
Daily laborer	24 5.0)
Private worker	15 (3.2)
Merchant	15 (3.2)
Government employee	6 (1.2)
Others	18 (3.8)

### Measurements

TB suspects were identified through a house-to-house survey by asking heads of the households for the presence of individuals with cough for at least two weeks. Data were collected by trained high school educated personnel using a pretested structured questionnaire in Amharic and Afaan Oromo. The questionnaire contained questions about socio-demographic characteristics, knowledge about TB, health care seeking behavior and stigma towards TB. The TB knowledge questions are summarized in Table 2. We also asked other close ended questions: Have you ever had cough for two or more weeks? If you had cough for two or more weeks, what did you do? Do you know a person with TB? Did you ever take anti-TB treatment? To assess stigma, we used 11 questions adopted from Somma and colleagues [17]. Each question consisted of four responses (strongly disagree, disagree, agree and strongly agree) where ‘strongly agree’ and ‘agree’ indicated presence of perceived stigma and ‘disagree’ and ‘strongly disagree’ indicated absence of perceived stigma. An answer consistent with stigma towards TB was scored with one point. An answer not consistent with stigma towards TB was scored as zero point. A total stigma score for TB was created by summing the scores of all questions. The stigma score ranged from 1 to 4, with the higher the score, the greater the degree of stigma towards TB. Individuals who had a stigma score of equal to or greater than the mean score of the study population were categorized as having high stigma towards TB. On the other hand, individuals who scored a stigma score below the mean were categorized as having low stigma towards TB. Since the stigma scores were normally distributed, the mean was used to classify the study population as having high or low stigma.

**Table 2:** Knowledge about TB among TB suspects (n = 395) in the Gilgel Gibe field research area, south west Ethiopia, March 2009.

Variable	Number (%)
**Perceived causes of TB**	
Evil eye	199 (50.4)
Germs	133 (33.7)
Satan or witchcraft	63 (15.9)
**Perceived mode of transmission of TB**	
Air borne (cough)	331 (83.8)
Unclean food and water	135 (34.2)
Being in a crowd	65 (16.5)
Sex with a person with TB	52 (13.2)
Consumption of uncooked milk	33 (8.4)
Other	58 (14.7)
**Perceived organ affected by TB**	
Lung	362 (91.6)
Intestine	35 (8.9)
Bone	33 (8.4)
Others	24 (6.1)
**Perceived symptoms of TB**	
Cough for 2 weeks or more	294 (74.4)
Hemoptysis	200 (50.6)
Fever for 2 weeks or more	152 (38.5)
Weight loss and fatigue	146 (37)
Night sweats	125 (31.6)
Shortness of breath	104 (26.3)
Chest pain	91 (23.0)
Loss of appetite	44 (11.1)
**TB is preventable disease**	
Yes	235(82.3)
No	34(8.6)
Do not know	36(9.1)
**TB transmission prevention methods**	
Avoiding cough in front of people	275(69.6)
Safe disposal of sputum	248(62.8)
Ventilation of living rooms	108(27.3)
Avoiding sex with TB patients	39(9.9)
**Heard about TB treatment**	
Yes	326(82.5)
No	69(17.5)

### Data analysis

Data were double entered using Epi-data version 3.1 (Epi-data, Norway, 2006). For analysis, the data were exported to SPSS version 15.0 statistical software (SPSS Inc. Chicago, 2007). Outcome variables (knowledge about TB, stigma towards TB and health seeking behavior) were dichotomized. Individuals who mentioned microorganism as a cause of TB were categorized as having ‘good’ knowledge. Those who mentioned other causes such as “evil eye” or Satan or witchcraft were categorized as having ‘poor’ knowledge. TB suspects who sought help in public or private health facilities were categorized as having ‘appropriate’ health seeking behavior and those who did nothing or visited other sources such as traditional healers were categorized as having ‘inappropriate’ health seeking behavior. Socio-demographic characteristics of the study participants were major independent variables. Stigma and knowledge were additional independent variables to represent health seeking behavior. The data analysis was based on logistic regression. First, the different independent variables were fitted univariately to assess their independent effect in terms of the crude odds ratio and its 95% confidence interval (CI). Next, a multivariable logistic regression model was fitted containing all the independent variables that showed a significant effect in the univariate analysis at the 5% significance level, leading to adjusted odds ratio and their 95% confidence intervals.

### Ethics statement

The study was approved by the ethical review committees of Jimma University, the Armauer Hansen Research Institute and Prince Leopold Institute of Tropical Medicine. Written consent was obtained from the study participants.

## Results

### Study participants characteristics

In a house-to-house survey of the 10,859 households in the study area, 476 (1.9% of adult population) TB suspects were identified from 410 households. They were all interviewed. There were 356 households with 1 TB suspect, 52 households with 2 TB suspects and 2 households with 3 TB suspects. One hundred and fifty-six (32.8%) TB suspects had cough for more than 8 weeks, 143 (30.0%) cough since 4–8 weeks and 177 (37.2%) for 2–3 weeks. Women (60.3%), married individuals (72.9%), Muslims (90.5%) and Oromo people (91.4%) constituted the majority of the study population. (**Table 1**).

### Knowledge

Three hundred ninety-five (83%) TB suspects had ever heard of TB. Only these 395 TB suspects are incorporated in the further analysis. Individuals who could read and write were more likely to have heard of TB [(crude OR = 2.98, (95%CI: 1.25, 7.08)] than those who could not read and write. “Evil eye” (50.4%), germs (33.7%), Satan and witchcraft (15.9%) were thought to be causes of TB. 91.6% of the TB suspects thought that the lungs were the most affected part of the body. Cough for more than 2 weeks (74.4%) and hemoptysis (50.6%) were mentioned as TB symptoms. Airborne transmission through coughing (83.8%), drinking unclean water and eating unclean food (34.2%) were stated as modes of TB transmission. Further, 82.3% responded that it is possible to prevent TB. To avoid coughing in front of people and proper disposal of sputum were cited as preventive strategies by 69.9% and 63.1% of the respondents respectively. 82.5% of the TB suspects have heard about TB treatment. (**Table 2**).

Individuals who could read and write were more likely to know that a microorganism is the cause of TB [(adjusted OR = 3.16, (95%CI: 1.77, 5.65)] than those who could not read and write. Males were more likely to know the cause of TB [(adjusted OR = 1.92, (95%CI: 1.22, 3.03)] compared to females. (**Table 3**). Individuals who could read and write were more likely to be aware of anti-TB drugs than individuals who could not read and write. The difference was not statistically significant though [(crude OR = 2.0, (95%CI: 0.92, 4.40)].

**Table 3:** Factors associated with knowledge about the cause of TB among TB suspects (n = 395) in the Gilgel Gibe field research area, south west Ethiopia, March 2009.

Variable		Cause of TB is a microorganism		Crude OR(95% CI)	Adjusted OR †(95% CI)
		Yes (%)	No (%)		
**Marital status**	Single	19 (48.7)	20 (51.3)	3.80 [1.08, 13.43]	2.08 [0.55, 7.95]
	Married	100 (34.2)	192 (65.8)	2.09 [0.68, 6.43]	2.00 [0.62, 6.51]
	Widowed	10 (22.7)	34 (77.3)	1.18 [0.32, 4.33]	1.64 [0.42, 6.38]
	Divorced	4 (20)	16 (80)	1	1
**Sex**	Male	71 (44.4)	89 (55.6)	2.23 [1.45, 3.41]	1.92 [1.22, 3.03]*
	Female	62 (26.4)	173 (73.6)	1	1
**Can read and write**	Yes	44 (57.9)	32 (42.1)	3.55 [2.12, 5.96]	3.16 [1.77, 5.65]*
	No	89 (27.9)	230 (72.1)	1	1

*Significant (*p*<0.05),

† Adjusted for marital status, sex and literacy status.

### Attitude and stigma

The instrument used in this study to asses TB stigma had a good internal consistency (Cronbach's α = 0.87). Of the 395 study participants who ever heard of TB, 5 did not have complete data on TB stigma questions. Only the 390 participants with complete data were included in the analysis. A large proportion of the TB suspects (51.3%) perceived that other people would think less of them if they knew they had TB, 39.5% would be embarrassed if they had TB, 30.3% thought that other people would avoid them if they had TB, 15.1% wanted to keep a possible diagnosis of TB concealed from a confidant. About one fifth thought that TB would have an effect on finding a partner for marriage, the willingness of the partner to have sex, and to be accepted as a member of a social group. (**Table 4**). Mean stigma score of the study population was 23.82 (minimum 11 and maximum 39). One hundred and ninety (48.8%) had low stigma and 199(51.2%) reported high stigma towards TB. Stigma was not associated with gender, knowledge about cause and treatment of TB, literacy status, religion, marital status, age, previous anti-TB treatment and previous exposure to a TB patient. (**Table 5**).

**Table 4:** Attitude of TB suspects (n = 390) towards TB in the Gilgel Gibe field research area, southwest Ethiopia, March 2009.

Stigma questions	Presence or absence of stigma for each stigma item	
	Present (%)	Absent (%)
If you had TB, others would think less of you	200 (51.3)	190 (48.7)
If you had TB, you would be ashamed/embarrassed	154 (39.5)	236 (60.5)
If you had TB, others would avoid you	118 (30.3)	272 (69.7)
If you had TB, you would have a problem of finding a partner for marriage even after cure	80 (20.5)	310 (79.5)
If you had TB, your partner would refuse to have sex with you	87 (22.3)	303 (77.7)
If you had TB, you would be asked to stay away from a social group	94 (24.1)	296 (75.9)
If you had TB, you would not disclose even to a confidant	59 (15.1)	331 (84.9)
If you had TB, you would think less of yourself	95 (24.4)	295 (75.6)
If you had TB, you would make others affected by the disease	33 (8.5)	357 (91.5)
If you had TB, others would think less of your family	165 (42.3)	225 (57.7)
If you had TB, it would be a problem for your children	273 (70.0)	117 (30.0)

**Table 5:** Factors associated with stigma towards TB among TB suspects (n = 389) in the Gilgel Gibe field research area, south west Ethiopia, March 2009.

		Stigma*		Crude OR (95% CI)
		High n(%)	Low n(%)	
**Sex**	Male	75(47.2)	84(52.8)	1.30[0.87, 1.97]
	Female	124(53.9)	106(46.1)	1
**Cause of TB**	Germ	63( 48.5)	67(51.5)	0.85[0.56, 1.30]
	Not germ	147(56.8)	112(42.2)	1
**Literate**	Yes	36(48.0)	39(52.0)	0.86[0.52, 1.42]
	No	177(56.8)	137(43.2)	1
**TB can be treated**	Yes	192(51.1)	184(48.9)	1
	No	7(53.8)	6(46.2)	1.12[0.37, 3.39]
**Religion**	Muslim	1181(51.9)	168(48.1)	1
	Orthodox	16(44.4)	20(55.6)	0.83[0.43, 1.60]
	Protestant	2(50.0)	2(50.0)	0.75[0.12, 4.53]
**Marital status**	Married	152(53.0)	135(47.0)	1
	Single	15(38.5)	24(61.5)	0.55[0.28, 1.02]
	Divorced	7(35.0)	13(65.0)	0.48[0.19, 1.23]
	Widowed	25(58.1)	18(41.9)	1.23[0.65, 2.36]
**Age**	15–34	59(48.4)	63(51.6)	1
	35–54	115(59.6)	78(40.4)	1.57[0.98, 2.49]
	>54	41(55.4)	33(44.6)	1.33[0.74, 2.37]
**Previous anti-TB treatment**	Yes	24(42.9)	32(57.1)	1.48[0.83, 2.62]
	No	175(52.6)	158(47.4)	1
**Know a person with TB**	Yes	90(50.8)	87(49.2)	0.98[0.66, 1.46]
	No	109(51.4)	103(48.6)	1

*High = total stigma score greater than or equal to the mean score of the study population, low =  total stigma score less than the mean score of the study population.

### Health care seeking behavior

Two hundred twenty (46.2%) TB suspects did not seek help, 120 (25.2%) contacted a health institution, 125 (26.3%) went to drug vendors, 29 (6.1%) did self medication and 2(0.4%) went to traditional healers. The overall median delay to seek help somewhere (except visits to traditional healers) was 4 weeks (range 2–52 weeks). Lack of money mainly for transportation (125 (56.8%)), the perception that the disease will improve (104 (47.3%)), considering the disease to be harmless (19 (8.6%)) and no health facility around (3 (1.4%)) were mentioned as reasons for not seeking help.

Individuals who had previous anti-TB treatment were more likely to take appropriate action for their illness [(adjusted OR = 3.65, (95%CI: 1.89, 7.06))] than individuals who had not experienced anti-TB treatment. Health seeking behavior was not associated with gender, literacy status, marital status, knowledge about cause of TB, awareness about anti-TB treatment, stigma, age, occupation and knowing a TB patient. (**Table 6**).

**Table 6:** Factors associated with health seeking behavior among TB suspects (n = 389) in the Gilgel Gibe field research area, south west Ethiopia, March 2009.

		Health seeking behavior#		Crude OR(95% CI)	Adjusted OR(95% CI)*
		Appropriate n(%)	Not appropriate n(%)		
**Sex¶**	Male	84(52.8)	75(47.2)	0.97[0.67, 1.41]	
	Female	115(50.0)	115(50.0)	1	
**Can read and write¶**	Yes	39(52.0)	36(48.0)	1.04[0.63, 1.73]	
	No	160(51.0)	154(49.0)	1	
**Marital status¶**	Single	23(59.0)	16(41.0)	2.88[0.95, 8.72]	
	Married	147(51.4)	139(48.6)	2.12[0.83, 5.40]	
	Widowed	22(51.2)	21(48.8)	2.10[0.71, 6.21]	
	Divorced	7(33.3)	14(66.7)	1	
**Cause TB¶**	Germ	66(50.8)	64(49.2)	0.98[0.64, 1.49]	
	Not germ	133(51.4)	126(48.6)	1	
**Heard about TB treatment**	Yes	172(53.6)	149(46.4)	1	1
	No	27(39.7)	41(60.3)	1.75[1.03, 3.0]	0.65[0.38, 1.13]
**Stigma¶**	High	108(54.3)	91(45.7)	0.78[0.52, 1.15]	
	Low	91(47.9)	99(52.1)	1	
**Age¶**	15–34	57(46.7)	65(53.3)	1	
	35–54	109(56.5)	84(43.5)	1.48[0.94, 2.33]	
	>54	33(44.6)	41(55.4)	0.92[0.51, 1.64]	
**Occupation¶**	Farmer	138(55.2)	112(44.8)	1	
	Housewives	23(41.8)	32(58.2)	0.58[0.32, 1.05]	
	Government employee	3(50)	3(50)	0.81[0.16, 4.10]	
	Daily laborer	5(23.8)	16(76.2)	0.25[0.90, 0.71]	
	Student	11(68.8)	5(31.2)	1.79[0.60, 5.29]	
	Merchant	9(69.2)	4(30.8)	1.83[0.55, 6.09]	
	Private work	5(35.7)	9(64.3)	0.45[0.15, 1.38]	
	Others	5(35.7)	9(64.3)	0.45[0.15, 1.38]	
**Previous anti-TB treatment**	Yes	43(76.8)	13(23.2)	3.87[2.01, 7.44]	3.65[1.89, 7.06]†
	No	156(46.8)	177(53.2)	1	1
**Know a person who had TB¶**	Yes	88(50)	88(50)	0.92[0.62, 1.37]	
	No	111(52.1)	102(47.9)	1	

#Appropriate =  Visits to health institutions, Not appropriate =  visits to sources other than health institutions.

¶Excluded from final model.

*Adjusted for information on anti-TB treatment and previous anti-TB treatment,

† Statistically significant (p<0.05).

## Discussion

In this study, we identified gaps in knowledge about the cause of TB, inappropriate health care seeking behavior and stigma towards TB. About 83% of TB suspects had heard of TB which is similar to a study done in north Ethiopia where 86% of the study participants were aware of TB [30] but lower than the 99.1% reported from India [31].

Traditional beliefs such as “evil eye”, Satan and witchcraft were the commonest perceived causes of TB in our study. In Tanzania, a significant number of people also mentioned that witchcraft could be the cause of TB [32]. Cold air, alcohol, smoking and lack of sanitation were common perceived causes in different studies [30], [32]. These traditional beliefs might contribute to the spread of TB as most people with such beliefs may not visit health facilities. A study from Ethiopia showed that 46% of patients seeking care at health facilities did so after informal treatment failed. Moreover, patients' poor perception of the cause of TB such as “evil eye” were related to a prolonged delay to seek medical care [18] although it was not statistically significant in the present study. In our study, only 33.7% of respondents knew that TB is caused by a microorganism which is higher than the finding in Vietnam (22%) [33]. Individuals who could read and write were more likely to know the cause of TB which is consistent with previous reports [30], [33]. Males were more likely to know the cause of TB compared with females. The poor knowledge among women and non-educated individuals concerning the cause of TB will result in inappropriate health care seeking behavior [34].

A significant number of study participants had perceived stigma of TB on marital prospects, social and sexual relationships. More than half of the respondents perceived that other people would consider them inferior and a third perceived that others would avoid them as a result of their illness. Such perception might have great impact on the social, psychological and mental well being of the victim and also his/her family. This could have dire consequences in a society where informal social organizations play a pivotal role in the daily lives of individuals. TB patients may deliberately conceal their status to avoid isolation. They may try to live with it for as long as possible, being the source of infection to others. The social isolation of TB patients was also described in Ghana [35] and Nepal [36]. In Ghana the community felt that TB patients should not sell their items in the market. In Nepal, there was a general believe that you should not meet with people who have TB and not visit a home where there is a household member with TB. In our study stigma was not influenced by gender and other socio-demographic variables. A multi-country study however showed that women had more impaired self esteem, social isolation and perceived stigma as compared to men [17].

The health care seeking behavior of our study participants was poor. The majority of them did not seek help for their illness as a result of wrong perceptions and lack of financial resources mainly for transport. A significant number of them did nothing since they considered that their illness was not severe. Similar reasons were mentioned in northwest Ethiopia [24], Vietnam [33] and China [37]. In our study, the health care seeking behavior was not affected by gender, the ability to read and write, marital status, knowledge about cause of TB, information about TB treatment, perceived stigma, age, occupation or familiarity with TB patient. But those who ever took anti-TB treatment were more likely to take appropriate action for their illness. Similar finding was reported from another study in North West part of Ethiopia [24]. In a study from Tanzania, perceived stigma was also not associated with a particular type of health care seeking behavior [38] but in other studies health care seeking behavior was affected by knowledge [19], [33], gender [23], [33], [39], [40] and education [25], [33].

In this study, we tried to assess several behavioral factors related to knowledge about and stigma towards TB in a rural community which could be potential barriers for national TB control program. However, the study was not without shortfalls. First, we didn't do focus group discussion to triangulate the findings. Second, the stigma questionnaire was not validated.

In conclusion, there was little knowledge about TB in the Gilgel Gibe field research area. We observed inappropriate health care seeking behavior and stigma towards TB. TB control programs in Ethiopia should educate rural communities, particularly females and non-educated individuals, about the cause and the importance of early diagnosis and treatment of TB.
